# Interleukin-1 as a Common Denominator from Autoinflammatory to Autoimmune Disorders: Premises, Perils, and Perspectives

**DOI:** 10.1155/2015/194864

**Published:** 2015-02-16

**Authors:** Giuseppe Lopalco, Luca Cantarini, Antonio Vitale, Florenzo Iannone, Maria Grazia Anelli, Laura Andreozzi, Giovanni Lapadula, Mauro Galeazzi, Donato Rigante

**Affiliations:** ^1^Interdisciplinary Department of Medicine, University of Bari, Piazza Giulio Cesare 11, 70124 Bari, Italy; ^2^Research Center of Systemic Autoimmune and Autoinflammatory Diseases, University of Siena, Viale Bracci 1, 53100 Siena, Italy; ^3^Institute of Pediatrics, Università Cattolica del Sacro Cuore, Largo Agostino Gemelli 8, 00168 Rome, Italy

## Abstract

A complex web of dynamic relationships between innate and adaptive immunity is now evident for many autoinflammatory and autoimmune disorders, the first deriving from abnormal activation of innate immune system without any conventional danger triggers and the latter from self-/non-self-discrimination loss of tolerance, and systemic inflammation. Due to clinical and pathophysiologic similarities giving a crucial role to the multifunctional cytokine interleukin-1, the concept of autoinflammation has been expanded to include nonhereditary collagen-like diseases, idiopathic inflammatory diseases, and metabolic diseases. As more patients are reported to have clinical features of autoinflammation and autoimmunity, the boundary between these two pathologic ends is becoming blurred. An overview of monogenic autoinflammatory disorders, PFAPA syndrome, rheumatoid arthritis, type 2 diabetes mellitus, uveitis, pericarditis, Behçet's disease, gout, Sjögren's syndrome, interstitial lung diseases, and Still's disease is presented to highlight the fundamental points that interleukin-1 displays in the cryptic interplay between innate and adaptive immune systems.

## 1. Introduction

Autoinflammatory and autoimmune diseases share many characteristics, starting with the prefix “auto” to define a pathological process directed against the* self*: they are systemic diseases, frequently involving multiple organs; both include monogenic and polygenic diseases, and both are characterized by immune system overactivity. However, the specific effectors of these disorders diverge, as the innate immune system directly causes tissue inflammation in the first, whereas dysregulation of both innate and adaptive immunity is operative in the latter. Mutations in the inflammasome-related genes have been associated with autoinflammation, and the role of this multiprotein-complex has been postulated also in organ-specific autoimmunity, since a wide spectrum of endogenous danger signals can activate inflammasome products, including interleukin-1*β* (IL-1*β*), and trigger adaptive immunity pathways.

Over the last decade, genome-wide association studies, which use single-nucleotide polymorphism arrays to identify genetic variants with pathogenetic effects in large patient populations, have been conducted for many autoimmune diseases, and new techniques such as high-throughput proteomics and exome sequencing are disclosing novel key-regulators of both immune tolerance and IL-1*β* biosynthesis.

The identification of novel genes and pathways driving human inflammatory diseases proceeds at an accelerated rhythm: so, what do we know for now?

## 2. Monogenic Autoinflammatory Disorders

The growing progress on cellular and molecular biology has revealed that an impaired control of innate immune system generates the so-called autoinflammatory disorders (AIDs), a group of heritable diseases characterized by unprovoked attacks of systemic inflammation in the absence of autoantibodies and autoreactive T cells [1, 2]. After the discovery of the familial Mediterranean fever-causing gene in 1997, we have witnessed an exciting revolution in the classification of monogenic AIDs with different genetic grounds and in our understanding of intrinsic mechanisms of inflammation. The unifying pathogenetic mechanism of AIDs relies in a lacking regulation of the inflammasome which leads to overproduction of proinflammatory cytokines, especially IL-1*β* [3]. The family of AIDs (briefly listed in Table 1) includes hereditary periodic fever syndromes and pyogenic and granulomatous disorders, all characterized by recurrent fever attacks accompanied by increase of acute-phase reactants and several overlapping clinical features, that is, rash, serositis, or arthritides usually starting in childhood [4–14]. The development of systemic amyloidosis, due to the deposition of a cleavage product, serum amyloid-A, one of the acute reactants produced during disease flares, is the deadly long-term complication of AIDs [15–17]. Since IL-1*β* plays a pivotal role in the pathogenesis of most AIDs, monotherapy blocking IL-1 activity results in a sustained reduction of disease severity, regardless of whether the therapeutic agent is anakinra, canakinumab, or rilonacept [18–21]. A checklist of papers dealing with anti-IL1 agents in AIDs is shown in Table 2, and the latest ongoing clinical trials can be found in Table 3.

## 3. PFAPA Syndrome

The periodic fever, aphthous stomatitis, pharyngitis, and cervical adenitis (or PFAPA) syndrome is an acquired disorder of unknown etiology characterized by periodically recurring episodes of high fever accompanied by at least one among aphthous stomatitis, pharyngitis, or cervical lymph node enlargement [129–132]. Disease onset is generally before 5 years, and persistence in adulthood has been described as well [133]. The exact pathogenesis of the syndrome remains enigmatic, though the brilliant response to single doses of corticosteroids [134] has led to the hypothesis that it might be an acquired autoinflammatory disorder with aberrant cytokine expression [135, 136]. Indeed, at the molecular level, data from Stojanov et al. have highlighted the role of the proinflammatory cytokine IL-1*β*, which was found elevated in PFAPA patients even between inflammatory attacks [137]. Kolly et al. have found an increased release of IL-1*β* from stimulated peripheral blood mononuclear cells of children with PFAPA syndrome during febrile episodes. Moreover, approximately 20% of them have been identified as carrying* NLRP3* gene variants, strengthening the hypothesis that inflammasome-related genes might be involved and activated in this condition [138]. A further proof for the role of IL-1*β* derives from clinical responsiveness to IL-1*β* inhibition: a small uncontrolled study has suggested that treatment with anakinra reduces the duration of acute flares in PFAPA patients [139], and an adult case of PFAPA syndrome refractory to conventional therapy who was responsive to anakinra has also been reported [140]. Since no completely satisfactory treatment options exist for PFAPA syndrome, IL-1 inhibition should be considered in the corticosteroid-resistant cases, especially if adults.

## 4. Rheumatoid Arthritis

A host of proinflammatory cytokines, namely, tumor necrosis factor (TNF-*α*), IL-1*β*, and IL-6, are involved in the pathogenesis of rheumatoid arthritis (RA) and are crucial to determine progression of chronic joint inflammation and concomitant bone erosion [141–143]. Serum and synovial concentrations of IL-1*β* have been found higher in patients with active RA than those in remission [144, 145]. Furthermore, several studies have shown that IL-1*β* induces the expression of different proteolytic enzymes, such as the metalloproteinases collagenase and elastase, resulting in destruction of the cartilaginous tissue. On this basis the understanding of RA pathophysiological mechanisms has clarified the role of IL-1*β* and led to the identification of new potential targets for biological therapy. In this regard anakinra, alone or in combination with methotrexate, has been evaluated in several controlled studies of patients with RA, revealing both decreased disease activity and decreased radiological progression of joint damage in the short term [146–166]. However, even though promising, anakinra seems less effective than other biologic agents in RA, like TNF-*α* inhibitors [167–170]. A phase II dose-finding study has investigated the favorable response of canakinumab in patients with active RA despite ongoing therapy at stable doses of methotrexate [171]. Integrated analysis from 37 phases II-III studies describing over 13.000 patients with RA showed that there was only a low probability that canakinumab would be better than the most effective current treatments [172].

## 5. Type 2 Diabetes Mellitus

Activation of the innate immune system has been shown relevant in the pathogenesis of type 2 diabetes mellitus (T2DM) [173], and caspase-1 dependent IL-1 production has been demonstrated in macrophages isolated from fat tissue of patients with T2DM [174]. High serum concentrations of glucose lead to increased IL-1*β* production in human *β*-cells, which is followed by NF-kB activation and Fas signalling upregulation, inducing *β*-cell apoptosis [175–177]. In addition, other authors have found that oligomers of islet amyloid polypeptide, a protein deposited in the pancreas of patients with T2DM, might trigger* NLRP3* inflammasome, enhancing mature IL-1*β* production and resulting in progressive decrease in *β*-cell number, followed by insulin resistance [178–181]. Based on the hypothetic role of IL-1*β* in the pathogenesis of T2DM, several studies were performed to prove that IL-1 blockade improved *β*-cell function and glycemic control. In this regard a double-blind clinical trial involving 70 patients with T2DM revealed that anakinra administration improves beta-cell secretory function: moreover, the beneficial effects on insulin production and systemic proinflammatory parameters were prolonged over time, even after anakinra cessation [182, 183]. Convincing evidence derives also from the positive effects on HbA1c levels of a single dose of canakinumab in T2DM [184–186]. A large phase III clinical trial CANTOS (Canakinumab Anti-inflammatory Thrombosis Outcomes Study) is currently recruiting participants to assess whether canakinumab may increase insulin secretion and insulin sensitivity in patients with T2DM (ClinicalTrials.gov NCT01327846). Gevokizumab has also proven to be useful in improving HbA1c [187–190]. Several clinical trials are in progress to determine whether gevokizumab might improve glycemic control in subjects with T2DM treated or not with metformin (ClinicalTrials.gov NCT01144975, NCT01066715, NCT00513214). Despite these encouraging results, further studies are needed to consider anti-IL1 agents as new therapeutic instruments for treating T2DM and probably a wider range of metabolic disturbances.

## 6. Uveitis

This sporadic disease has a baffling etiology and is the most frequent extra-articular sign of different systemic autoimmune rheumatologic disorders, such as oligoarticular variant of juvenile idiopathic arthritis, seronegative spondyloarthritis, and Behçet's disease [191]. The inflammatory process leading to uveitis is mainly driven by Th17 cells and sustained by intricate sceneries directed by many proinflammatory cytokines, chiefly TNF-*α* and IL-1*β* [192]. In this regard Kitamei et al. have suggested that NF-*κ*B, activated by IL-1*β*, plays a pivotal role in an experimental murine model of autoimmune uveoretinitis: in fact, pyrrolidine dithiocarbamate administration, which inhibits NF-*κ*B signaling, has ameliorated clinical symptoms and suppressed ocular IL-1*β* mRNA expression [193]. Lennikov et al. have shown that uveitis improves after inhibition of I*κ*B kinase *β* in an animal model of endotoxin-induced uveitis, particularly when the disease is due to IL-1*β* and TNF-*α* oversecretion [194]. Several years before other authors had demonstrated that foot-pad injection of lipopolysaccharide in rats induced elevated mRNA expression of various cytokines, such as TNF-*α*, IL-1*β*, and IL-1 receptor antagonist (IL-1Ra) in the uvea and retina, suggesting that these mediators contribute to the development and recovery of ocular inflammation [195, 196]. These evidences have also been corroborated by the capability of anakinra to suppress immune-mediated ocular inflammation not only in animal models [197] but also in a patient with Blau syndrome and in a patient with CINCA syndrome [73, 198]. More recently a 4-year-old boy diagnosed with early onset sarcoidosis and presenting with refractory severe panuveitis has experienced a rapid remission of uveitis and normalization of most gene expression profiles following canakinumab administration [118]. In addition, after the results of the previous study conducted by Gül et al. on Behçet's disease-resistant uveitis [199], three multicenter phase III clinical trials are ongoing to test safety and efficacy of gevokizumab in the treatment of active noninfectious uveitis (ClinicalTrials.gov NCT01684345), quiescent noninfectious uveitis (ClinicalTrials.gov NCT01747538), and Behçet's disease-associated uveitis (ClinicalTrials.gov NCT01965145). In the end, a phase II clinical trial is also being conducted with gevokizumab in patients with active scleritis (ClinicalTrials.gov NCT01835132).

## 7. Pericarditis

Acute pericarditis may be the first manifestation of an underlying systemic disease [200–203]. Although a specific exact etiology may be identified, its cause remains obscure in up to 85% of patients. Idiopathic recurrent acute pericarditis (IRAP) is a troubling complication of acute pericarditis, occurring in approximately 30% of cases [204]. Recently, considering clinical and laboratory features of IRAP (absence of autoantibodies or self-antigen-specific T lymphocytes) [205–208] and the growing evidence about IRAP favourable response to IL-1 inhibition, it has been hypothesized that this condition can be included in the group of AIDs [209–211]. However, IRAP may occur in the framework of two peculiar AIDs, familial Mediterranean fever (FMF) and TNF receptor-associated periodic syndrome (TRAPS), becoming a diagnostic clue for identifying these disorders [22, 59, 212–214]. IRAP may also be the only clinical TRAPS symptom in patients carrying low-penetrance* TNFRSF1A* mutations [215–217]. According to some authors this recurrent pericarditis can be considered an example of a multifactorial disorder with overlapping pathogenic mechanisms, both autoinflammatory and autoimmune [218]. On this basis, a multicentre study evaluating the incidence of TRAPS mutations in patients with IRAP has demonstrated that positive family history of pericarditis, pericarditis recurrence, failure of treatment with colchicine, and need of immunosuppressive agents were key-elements for suspecting a clinical diagnosis of TRAPS [219, 220]. Due to these findings, FMF and above all TRAPS should be taken into account in the differential diagnosis of IRAP [221]. Treatment with anakinra has been described as dramatically effective in the control of IRAP, even the corticosteroid-dependent forms of IRAP or those resistant to conventional therapies [210, 211, 222, 223]. Additional data derive from the study of 15 young patients with IRAP performed by Finetti et al., who evaluated long-term use of anakinra: after a median follow-up of 39 months a 95% reduction of flares was observed, and patients experienced a persistent disease control [209]. More recently Lazaros et al. have confirmed that anakinra is highly effective also in adults with IRAP [224]. A phase IV study designed to demonstrate the efficacy of anakinra in patients with IRAP is actually ongoing (ClinicalTrials.gov NCT02219828), but there are no data about treatment with rilonacept, canakinumab, and gevokizumab.

## 8. Behçet's Disease

The pathogenesis of Behçet's disease (BD) is still largely unknown, and continuous efforts are in progress to characterize its biologic background, suggesting that the disease may lie at the crossroad between autoinflammatory and autoimmune syndromes [1, 225]. The central role of innate immunity in its pathogenesis has been suggested not only by the increased levels of IL-1 in serum [226] and synovial fluid [227] of BD patients but also by the beneficial effects obtained with IL-1 inhibition [228]. In addition, the time of disease onset has been correlated with IL-1 gene specific single nucleotide polymorphisms [229, 230]. Recently Castrichini et al. have observed an increased expression of the P2X7 receptor, a nucleotide-gated ion channel, in BD monocytes, acting in the promotion of IL-1*β* release [231]. With regard to therapy, the efficacy of anakinra on various BD manifestations has been well documented in some surveys and several case reports, albeit with variable duration of response [232–236]. A pilot study is ongoing to test whether anakinra given at a daily dose of 100 mg with a dose escalation up to 200 mg/day might control all BD manifestations (ClinicalTrials.gov NCT01441076). The favorable response to anti-IL-1 agents has been also confirmed in three adult BD patients who received canakinumab (150 mg every 6 weeks) as monotherapy [237] and in other two cases [228, 238].  Table 4 shows a list of papers dealing with IL-1 blockade in BD. Additional convincing evidence for a possible role of IL-1*β* in BD emerges from a trial with the monoclonal anti-IL-1*β* antibody gevokizumab, which has proven to be effective in both uveitis and retinal vasculitis, leading to complete resolution of intraocular inflammation [199]. These findings have represented an important aspect in the management of BD, revealing that IL-1 inhibition can be indicated in the treatment of the most severe ocular manifestations, especially if unresponsive to immunosuppressant drugs and other biologicals [239–241].

## 9. Gout and Chondrocalcinosis

Proinflammatory cytokines have a nodal role in orchestrating the body reaction to monosodium urate (MSU) and calcium pyrophosphate dihydrate crystals: recent attention has been focused on the role of IL-1 [242]. Experimental models have suggested that crystals engage the caspase-1-activating* NLRP3* inflammasome, resulting in the production of bioactive IL-1*β* [243]. Further evidence for the proposed role of IL-1*β* in the pathogenesis of gout is shown by the study of Chen et al., in which they demonstrated that IL-1*β* receptor-deficient mice were not susceptible to MSU-induced inflammation [244]. Other authors have also assessed that activation of the P2X7R-mediated signalling pathway by MSU crystals leads to enhanced* NLRP3* inflammasome activity and IL-1*β* oversecretion at the onset of an acute gouty attack [245]. Interestingly, Owyang et al. have reported that gevokizumab was able to reduce acute inflammation in a mouse model, blocking MSU crystal-induced peritonitis [246]. Further proofs of the concept that IL-1 is clearly involved derive from the favorable results obtained with anakinra in an open-label study and several gout-related case reports [247–252]. In line with these findings, Vitale et al. have reported three patients with chronic tophaceous gout unresponsive to standard therapy, in whom anakinra led to remarkable amelioration of joint symptoms within 24 hours. Interestingly, patients were also affected by T2DM and, along with amelioration of joint symptoms, they also experienced a marked improvement in the glycemic control during anakinra treatment [253]. Also canakinumab and rilonacept have proven their efficacy on a broad sample of patients with crystal-induced arthritis [254–261], showing a superior therapeutic effect in comparison with corticosteroids. A phase III study testing efficacy of canakinumab in preventing gout relapses is now ongoing for patients with colchicine intolerance (ClinicalTrials.gov NCT01362608). As a result, anti-IL-1 agents should be taken into consideration for both patients affected with unresponsive gouty arthritis and those presenting with dysmetabolic comorbidities.

## 10. Sjogren's Syndrome

This autoimmune disorder is characterized by infiltration of mononuclear cells in the salivary and lacrimal glands, leading to dryness of both mouth and eyes. Although pathogenesis is not completely understood, several studies focusing on cytokine profile that may contribute to the pathological scenery of this disease [262, 263] have found an increased concentration of IL-1 in the salivary fluid and peripheral blood of patients with Sjogren's syndrome (SS), indicating that IL-1 works as a pivotal regulator in the development of local and systemic manifestations [264, 265]. Functional consequences of IL-1 and low levels of IL-1Ra in the saliva remain unclear. In this regard Dubost et al. have suggested that the salivary IL-1/IL-Ra imbalance may promote inflammatory lesions in the mouth [266], while Solomon et al. have shown that patients with SS produce higher concentration of IL-1*α* and IL-1*β* in the tears [262]. In addition, IL-1 expression in ocular epithelial cells has been correlated with keratinizing squamous metaplasia, a condition resulting from uncontrolled ocular inflammation [267]. Some authors have also found that IL-1*β* is involved in the destruction of salivary and lacrimal glands [268]: in fact, IL-1*β* may have a proteolytic activity, leading to acinar and ductal structure disruption in salivary glands of patients with SS [263]. Since acinar cells, duct cells, and blood vessels of the lacrimal glands are innervated by parasympathetic and sympathetic nervous system, several reports have shown that exogenously added IL-1 might inhibit neurotransmitter release [269–271]. Additional data substantiating the crucial role of innate immunity in SS derive from studies on nonobese autoimmune-prone mice. Bulosan et al. have investigated the potential involvement of inflammatory caspases, revealing a concurrent upregulation of caspase-11 in macrophages [272]. Moreover, the presence of the purinergic P2X7 receptor, an ATP-gated ion channel, in the salivary glands, would be capable of determining* NLRP3* inflammasome activation, leading to the release of mature IL-1*β* and IL-18 [273–275]. Since IL-1 seems to be directly involved in the pathogenesis of SS, there might be a rationale for using anti-IL-1 agents as a potential treatment [276]. In light of this evidence, a randomized double-blind placebo-controlled trial has indicated that IL-1 inhibition with anakinra is able to influence favorably fatigue in patients with SS [277]. More recently, data from a prospective double-blind randomized trial have also demonstrated that targeting IL-1 by topical application of anakinra is effective in reducing dry eye disease-related symptoms and corneal epitheliopathy [278].

## 11. Interstitial Lung Diseases

Chronic interstitial lung diseases (ILDs), characterized by diffuse lung interstitial wall inflammation, often result in severe pulmonary fibrosis and impaired gas exchange [279]. Alveolar macrophages are involved in various pulmonary inflammatory processes and can constitutively release IL-1, probably due to various exogenous and endogenous stimuli [280]. However, their activity is limited by the presence of IL-1 inhibitory factors secreted from the same macrophages [281]. On this basis, an imbalance between the release of IL-1 and its inhibitor may evoke an inflammatory state [282]. Several studies have revealed the presence of IL-1*β* in the chronically inflamed lung tissues undergoing fibrogenesis, suggesting a causative link between IL-1 and fibrosis [283–285]. Through IL-1*β* overexpression, induced by intratracheal administration of adenoviral genes, Kolb et al. have caused an acute pulmonary inflammation with severe progressive tissue fibrosis in an animal model [286]; they also found that IL-1*β* led to increased concentrations of growth factors in the bronchoalveolar lavage fluid [287]. Interestingly, IL-1 antagonists have been successfully used to block fibrosis in murine models of ILDs [288, 289]. A genetic variability in the* IL1RN* gene, encoding the physiological IL-1Ra, may contribute to the pathogenesis of idiopathic pulmonary fibrosis [290, 291]. Other authors have suggested that* NLRP3* inflammasome is involved in the early inflammatory process of pneumoconiosis and systemic sclerosis [292, 293]. In this regard a phase I/II study is ongoing to test the effect of the IL-1 inhibitor rilonacept on skin gene expression of patients with systemic sclerosis (ClinicalTrials.gov NCT01538719). Moreover, a case of antisynthetase syndrome clinically characterized by progressive and diffuse interstitial lung disease and myositis responding to anakinra has been reported [294], while another one lost its efficacy on lung disease manifestations [295]. Although IL-1*β* might be considered a valid target for treatment of ILDs, further studies are required to fully explore and define its exact role in the pathogenesis of ILDs.

## 12. Still's Disease

This rare inflammatory disorder of undisclosed etiology is characterized by fever, rash, arthritis, and prominent neutrophilia, accompanied by high C-reactive protein and ferritin levels [296]. Similarly to what we observe in the monogenic AIDs, the main proinflammatory cytokine increased in adult onset Still's disease (AOSD) is IL-1*β* [297, 298]. Macrophage activation syndrome has been rarely reported in the course of AOSD, but mortality in adults is far higher than that for children with systemic-onset juvenile idiopathic arthritis [299]. Convincing evidence about IL-1 involvement in this disorder derives from the study by Pascual et al., reporting that peripheral blood mononuclear cells of healthy subjects incubated with sera from patients with systemic-onset juvenile idiopathic arthritis secreted large amounts of IL-1*β* and led to increased expression of innate immunity genes [300]. Benefits obtained with IL-1 antagonists have also reinvigorated the concept that IL-1*β* is largely implicated in the pathological scenery of AOSD. Notably, anakinra as monotherapy has proven to be highly effective in patients refractory to conventional treatments (corticosteroids and methotrexate). These findings rely not only on single case reports and small case series [301–321] but also on large numbers of subjects: a study of 25 patients with active multiresistant AOSD reported a complete resolution of clinical activity in 84% of cases and normalization of laboratory markers in 80% [322]. Moreover, an open randomized multicentre study has enrolled 22 patients with AOSD, demonstrating that anakinra brings about remission in the refractory forms of the disease [323]. A recent retrospective study carried out to assess long-term efficacy of anakinra in 28 patients has shown a complete remission in 57% of them after a mean follow-up time of 23 months [324]. Also canakinumab and rilonacept have proven the efficacy of IL-1 inhibition in AOSD. In this regard Henderson et al. have investigated treatment with rilonacept in a small sample of patients, observing a good clinical response [325]. More recently the successful use of rilonacept in the management of three patients with refractory AOSD [326] as well as the effectiveness of canakinumab in curbing AOSD manifestations has been reported [327, 328]. A phase II study is now ongoing to assess whether canakinumab may confirm its promising effects in the decrease of disease activity (ClinicalTrials.gov NCT02204293).

## 13. Conclusions

From a mere pathogenic point of view most autoinflammatory and autoimmune diseases share the chronic aberrant activation of the immune system, which leads to tissue inflammation and/or tissue damage of varying magnitude and extent in genetically predisposed individuals. The specific effectors of inflammation and damage are different in the two groups of disorders, respectively, the innate and adaptive immunity branches, even if in the last decade we began to recognize the involvement of autoinflammatory circuits in many different diseases and also those having an autoimmune basis. Certainly, the role of IL-1 on lymphocyte function, favouring the expansion of autoreactive Th1 and Th17 cells or downregulating regulatory T cells, has not been yet completely elucidated and requires further research to change our way of categorizing an expanding group of inflammatory disorders, even autoimmune diseases.  Table 5 recapitulates all cells and the different biologic platforms involved in the various clinical settings driven by IL-1 oversecretion. Unearthing the molecular pathways of autoinflammation and autoimmunity has enlightened our capacity of understanding the human disease, and recent technological breakthroughs have also generated large quantities of novel information specifically in both autoinflammatory and autoimmune diseases at the genetic, transcriptional, proteic, and metabolic levels. The acceleration of clinical trials over the past decades has also included rare diseases, such as AIDs. Studies of such complex disorders and their relationship with IL-1 need to address heterogeneity in the human population, interaction with the environment, and effects of treatments, while a multidisciplinary approach should be ultimately fostered to provide a significant change in the knowledge of these diseases across the scientific community.

## Figures and Tables

**Table 1:** Brief summary of the monogenic autoinflammatory disorders.

Disease		*Gene* locus	Protein	Inheritance	Clinical features	Treatment
FMF [22, 23]		*MEFV* 16p13.3	Pyrin	AR	Fever, serositis, arthralgias or arthritides, erysipelas-like eruption on the legs, responsiveness to colchicine prophylaxis, amyloidosis in untreated or noncompliant patients	Colchicine, anakinra, canakinumab

TRAPS [24–30]		*TNFRSF1A* 12p13	p55 tumor necrosis factor receptor type 1	AD	Fever, severe migrating muscle and joint involvement, conjunctivitis, periorbital edema, arthralgias or arthritis, sacroiliitis, serosal involvement, steroid responsiveness of febrile attacks, risk of amyloidosis	Corticosteroids, etanercept, anakinra, canakinumab, tocilizumab

MKD [31, 32]		*MVK* 12q24	Mevalonate kinase	AR	Fever, widespread polymorphous rash, arthralgias, abdominal pain, diarrhea, lymph node enlargement, oral aphthosis	NSAIDs, anakinra, corticosteroids

CAPS [33, 34]	FCAS	*NLRP3* 1q44	Cryopyrin	AD	Fever, cold-induced urticaria-like rash, conjunctivitis, arthralgias, fatigue	Anakinra, canakinumab, rilonacept
	MWS				Fever, urticaria-like rash, conjunctivitis, arthralgias, neurosensorial deafness, risk of amyloidosis	
	CINCAs				Fever, urticaria-like rash, uveitis, papilledema, deforming arthritis involving large joints, neurosensorial deafness, aseptic chronic meningopathy, risk of amyloidosis	

PAPAs [35–37]		*PSTPIP1* 15q24-q25.1	CD2BP1	AD	Pauciarticular pyogenic arthritis, osteocartilaginous erosions of joints, cystic acne, pyogenic abscesses	Infliximab, anakinra

MAJEEDs [38–40]		*LPIN2* 18p11.31	Lipin 2	AR	Recurrent multifocal osteomyelitis, congenital dyserythropoietic anemia, chronic dermatosis resembling Sweet's syndrome	NSAIDs, corticosteroids, anakinra, canakinumab

BS [41–43]		*NOD2* *(CARD15)* 16q12.1-13	Nod2 (Card15)	AD	Intermittent fevers, granulomatous dermatitis with ichthyosis-like changes, symmetrical granulomatous polyarthritis, recurrent severe granulomatous panuveitis	Corticosteroids, immunosuppressive agents, anti-TNF-*α* drugs, anakinra

AD: autosomal dominant; AR: autosomal recessive; BS: Blau syndrome; CAPS: cryopyrin-associated periodic syndromes; CINCAs: chronic inflammatory neurological cutaneous articular syndrome; FCAS: familial cold autoinflammatory syndrome; FMF: familial Mediterranean fever; MAJEEDs: Majeed syndrome; MKD: mevalonate kinase deficiency syndrome; MWS: Muckle-Wells syndrome; NSAIDs: nonsteroidal anti-inflammatory drugs; PAPAs: pyogenic arthritis-pyoderma gangrenosum-acne syndrome; TRAPS: tumor necrosis factor receptor-associated periodic syndrome.

**Table 2:** Overview of the medical literature regarding anti-interleukin-1 therapies in the monogenic autoinflammatory disorders.

	FMF	TRAPS	MKD	CAPS	PAPAs	MAJEEDs	BS
Anakinra	[44–58]	[59] [60–64]	[65–72]	[73] [74–94]	[95–100]	[38]	[101, 102]

Canakinumab	[46, 50] [58, 103, 104]	[105–107]	[68]	[93, 94, 108–116]	[117]	[38]	[118]

Rilonacept	[50] [119–121]			[122–128]			

BS: Blau syndrome; CAPS: cryopyrin-associated periodic syndromes; FMF: familial Mediterranean fever; MAJEEDs: Majeed syndrome; MKD: mevalonate kinase deficiency syndrome; PAPAs: pyogenic arthritis-pyoderma gangrenosum-acne syndrome; TRAPS: tumor necrosis factor receptor-associated periodic syndrome.

**Table 3:** Recent and ongoing clinical trials on interleukin-1 blockade in the monogenic autoinflammatory disorders.

	Phase	Status	Study	Disease	ClinicalTrials.gov identifier
Anakinra	3	Recruiting	Kineret (anakinra) in adult patients with colchicine-resistant familial Mediterranean fever	FMF	NCT01705756
	1	Completed	The use of Kineret (anakinra) in the treatment of familial cold autoinflammatory syndrome	FACS	NCT00214851

Canakinumab	3	Recruiting	Efficacy, safety, and tolerability of ACZ885 in pediatric patients with the following cryopyrin-associated periodic syndromes: familial cold autoinflammatory syndrome, Muckle-Wells syndrome, and CINCA syndrome	CAPS	NCT01576367
	3	Completed	The safety and efficacy of canakinumab in patients aged 4 years or older diagnosed with cryopyrin-associated periodic syndromes in Canada	CAPS	NCT01105507
	3	Completed	Efficacy and safety study of canakinumab administered for 6 months (24 weeks) in Japanese patients with cryopyrin-associated periodic syndromes followed by an extension phase	CAPS	NCT00991146
	2	Completed	Evaluation of the safety and efficacy of canakinumab in pediatric patients with colchicine-intolerant or colchicine-resistant familial Mediterranean fever	FMF	NCT01148797
	2	Completed	Efficacy and safety of canakinumab in patients with colchicine-resistant familial Mediterranean fever	FMF	NCT01088880
	3	Active, not recruiting	Efficacy, safety, and tolerability of ACZ885 in pediatric patients with the following cryopyrin-associated periodic syndromes: familial cold autoinflammatory syndrome, Muckle-Wells syndrome, and CINCA syndrome	CAPS	NCT01302860
	—	Recruiting	Clinical outcomes and safety: a registry study of Ilaris (canakinumab) patients (B-confident)	CAPS	NCT01213641
	2	Active, not recruiting	Canakinumab in patients with active hyper-IgD syndrome	MKD	NCT01303380
	2	Completed	Efficacy and safety study of ACZ885 in patients with active recurrent or chronic tumor necrosis factor receptor-associated periodic syndrome	TRAPS	NCT01242813
	3	Recruiting	Efficacy and safety of canakinumab in patients with hereditary periodic fevers	HPFs	NCT02059291

CAPS: cryopyrin-associated periodic syndromes; FACS: familial cold autoinflammatory syndrome; FMF: familial Mediterranean fever; HPFs: hereditary periodic fever syndromes; MKD: mevalonate kinase deficiency syndrome; TRAPS: tumor necrosis factor receptor-associated periodic syndrome.

**Table 4:** Overview from the medical literature dealing with interleukin-1 blockade in Behçet's disease.

First author [reference]	Number of patients	Brief summary of clinical and laboratory features	Anti-interleukin-1 agents	Outcome
Botsios [235]	1	Fever, mucosal involvement, gut ischemic perforation, positive pathergy test, increased acute-phase reaction	Anakinra	Complete remission with improvement of inflammatory markers in 7–10 days

Bilginer [234]	1	Fever, mucosal involvement, erythema nodosum, arthritis, secondary amyloidosis, increased acute-phase reaction, skin pathergy reactions, overlap with familial Mediterranean fever	Anakinra	Complete remission with improvement of inflammatory markers in 6 months

Gül [199]	7	Acute posterior uveitis and panuveitis, retinal vasculitis	Gevokizumab	Complete remission of retinal vasculitis in 4–21 days and marked reduction in intraocular inflammation

Ugurlu [238]	1	Mucosal involvement, erythema nodosum, bilateral panuveitis, retinal vasculitis, skin pathergy reactions	Canakinumab	Complete remission for 8 weeks

Emmi [236]	1	Mucosal and gastrointestinal involvement, arthritis, pseudofolliculitis, bilateral retinal vasculitis	Anakinra	Complete remission after 12 months of follow-up

Cantarini [232]	9	Fever, mucosal involvement, headache, skin lesions, retinal vasculitis, low back pain, increased acute-phase reaction, arthritis, abdominal pain,	Anakinra	Complete/partial remission with a variable duration of response

Caso [233]	1	Mucosal and ocular involvement, pseudofolliculitis, sacroiliitis increased acute-phase reaction	Anakinra	Complete remission in few days

Cantarini [228]	1	Fever, mucosal involvement, skin lesions, arthritis, abdominal pain, headache, increased acute-phase reaction, overlap with granuloma annulare	Canakinumab	Complete remission after few weeks

Vitale [237]	3	Fever, mucosal and gastrointestinal involvement, headache, anterior uveitis, arthralgia, pseudofolliculitis, deep venous thrombosis, panuveitis, headache, arthritis, increased acute-phase reaction	Canakinumab	Complete remission within few weeks

**Table 5:** Main scene players involving the role of interleukin-1 in the autoinflammatory and autoimmune disorders described in this review.

Disease [reference]	Cells involved	Biologic platforms involved	Treatment [reference]
PFAPA syndrome [137, 138]	Mononuclear cells, neutrophils, lymphocytes, Th1 cells	*NLRP3* inflammasome	Anakinra [139, 140]

Rheumatoid arthritis [141–143]	T and B lymphocytes, plasma cells, synoviocytes	Metalloproteinases (collagenase, elastase)	Anakinra [167, 168], canakinumab [171, 172]

Type 2 diabetes mellitus [173–181]	Macrophages, adipocytes, pancreatic *β*-cells	Oligomers of islet amyloid polypeptide, *NLRP3* inflammasome	Anakinra [182, 183, 253], canakinumab [184–186], gevokizumab [187–190]

Uveitis [192–194]	Th17 cells	*NLRP3* inflammasome	Anakinra [73, 198], canakinumab [118], gevokizumab [199]

Pericarditis [22, 59, 212–218]	Dendritic cells, Th1 and Th17 cells, macrophages	Pyrin, p55 tumor necrosis factor receptor type 1	Anakinra [209, 224]

Behçet's disease [226–231]	Monocytes/macrophages, Th1 and Th2 cells, neutrophils	P2X7 receptor, *NLRP3* inflammasome	Anakinra [232–236], canakinumab [228, 237, 238]

Gout and chondrocalcinosis [242–245]	Neutrophils, macrophages	Lysosomal and cytoplasmic enzymes, P2X7 receptor, *NLRP3* inflammasome	Anakinra [247–253], canakinumab [254–257], rilonacept [258–261]

Sjögren syndrome [262–266, 272–275]	Mononuclear cells, Th1 and Th17 cells	Upregulation of caspase-11, STAT-1 activity, P2X7 receptor, *NLRP3* inflammasome, IL-1/IL-Ra imbalance	Anakinra [277, 278]

Interstitial lung diseases [280, 281]	Alveolar macrophages, neutrophils, macrophages	*NLRP3* inflammasome, IL-1/IL-Ra imbalance, transforming growth factor-*β*1	Anakinra [294, 295]

Still's disease [300]	Macrophages, neutrophils, natural killer cells, Th1 and Th17 cells, dendritic cells	*NLRP3* inflammasome	Anakinra [301–324], canakinumab [327, 328], rilonacept [325, 326]
